# Measuring a dynamical topological order parameter in quantum walks

**DOI:** 10.1038/s41377-019-0237-8

**Published:** 2020-01-20

**Authors:** Xiao-Ye Xu, Qin-Qin Wang, Markus Heyl, Jan Carl Budich, Wei-Wei Pan, Zhe Chen, Munsif Jan, Kai Sun, Jin-Shi Xu, Yong-Jian Han, Chuan-Feng Li, Guang-Can Guo

**Affiliations:** 1 CAS Key Laboratory of Quantum Information, University of Science and Technology of China, Hefei, 230026 China; 2 CAS Center for Excellence in Quantum Information and Quantum Physics, University of Science and Technology of China, Hefei, 230026 China; 3 Max-Planck-Institut für Physik komplexer Systeme, Nöthnitzer Straße 38, D-01187 Dresden, Germany; 4 Institute of Theoretical Physics, Technische Universität Dresden, 01062 Dresden, Germany

**Keywords:** Single photons and quantum effects, Quantum optics, Single photons and quantum effects, Ultrafast photonics

## Abstract

Quantum processes of inherent dynamical nature, such as quantum walks, defy a description in terms of an equilibrium statistical physics ensemble. Until now, identifying the general principles behind the underlying unitary quantum dynamics has remained a key challenge. Here, we show and experimentally observe that split-step quantum walks admit a characterization in terms of a dynamical topological order parameter (DTOP). This integer-quantized DTOP measures, at a given time, the winding of the geometric phase accumulated by the wavefunction during a quantum walk. We observe distinct dynamical regimes in our experimentally realized quantum walks, and each regime can be attributed to a qualitatively different temporal behavior of the DTOP. Upon identifying an equivalent many-body problem, we reveal an intriguing connection between the nonanalytic changes of the DTOP in quantum walks and the occurrence of dynamical quantum phase transitions.

## Taking stock of a quantum walk

A model describing the random walks of quantum particles has been developed by researchers in China and Germany. Classical phenomena such as molecules moving in gases or animals foraging for food can be described by random walks, where every step is chosen through processes like tossing a coin. For quantum particles, randomness arises from the transitions and entanglement of quantum states, but it is difficult to describe the emerging statistical patterns in these quantum walks. Chuan-Feng Li at the University of Science and Technology of China, Hefei, and co-workers used an experimental setup for observing the quantum walks of single photons. They found that the walks could be characterized by a so-called dynamical topological order parameter that describes the behavior of the particle’s wavefunction during the walk, thereby linking quantum effects to physical spatial measurements.

## Introduction

Coherence in quantum dynamics is at the heart of fascinating phenomena beyond the realm of classical physics, such as quantum interference effects^1^, entanglement production^2,3^ and geometric phases^4–6^. However, the identification of the general principles behind the inherent nonequilibrium nature of unitarily evolved quantum states still invokes central open questions^7^, which we experimentally address in the context of quantum walks below^8^. Quantum walks provide a powerful and flexible platform to experimentally realize and probe coherent quantum time evolution far from thermal equilibrium. As opposed to classical random walks, quantum walks are characterized by quantum superpositions of amplitudes rather than classical probability distributions. This genuine quantum character has already been harnessed in various fields of physics, ranging from the design of efficient algorithms in quantum information processing^9–11^, the observation of correlated dynamics^12–19^ and Anderson localization^20,21^ to the realization of exotic physical phenomena in the context of topological phases^22–38^. While the topological order can be retrieved in real space^39,40^, accessing the full complex amplitude information characterizing a coherent superposition remains one of the key challenges in quantum walk experiments.

In this work, we report the direct observation of a dynamical topological order parameter (DTOP) that provides a dynamical characterization of quantum walks. To this end, we realize a split-step quantum walk in a photonic system using the framework of time multiplexing. Using a previously developed technique, we achieve full-state tomography of the time-evolved quantum state for up to 10 complete time steps. Importantly, this measurement provides us with the full complex amplitude information of the quantum walk state. This information is essential for our central goal of a dynamical classification of a quantum walk using the DTOP, since the DTOP measures the phase winding number *ω*_*D*_(*t*) in momentum space, namely, of the so-called Pancharatnam geometric phase (PGP)^41,42^. From our measurements, we find that dynamical transitions between topologically distinct classes of quantum walks can be uniquely distinguished experimentally by the observed time-dependent behavior of *ω*_*D*_(*t*): For a quench between two systems with the same topological character, we find *ω*_*D*_(*t*) = 0 for all time steps; however, for a quench between two topologically different systems, *ω*_*D*_(*t*) also starts at *ω*_*D*_(*t* = 0) = 0 but monotonously changes at certain critical times. Generalizing these observations, we establish a unique relation between the behavior of *ω*_*D*_(*t*) and the change over a parameter quench in the topological properties of an effective Floquet Hamiltonian that stroboscopically describes the quantum walk.

While the quantum walk in our experiment realizes the dynamics of a single quantum particle, we establish an underlying many-body context that explains the points at which the DTOP *ω*_*D*_(*t*) changes nonanalytically in terms of a dynamical critical phenomenon. To this end, we map the superposition of Bloch waves realized in the quantum walk to a product state of a corresponding fermionic many-body system. Therefore, an intriguing analogy between our present experiment and the notion of dynamical quantum phase transitions (DQPTs) occurring in the unitary evolution of the quenched many-body system is revealed. Our work provides a dynamical characterization of the bulk topological properties and therefore complements the recent measurement of topologically protected boundary modes in quantum walks^24^, thus providing an important step toward a comprehensive understanding of the role of topology in quantum dynamics.

## Results

### Quantum walk setup

**Fig. 1:**
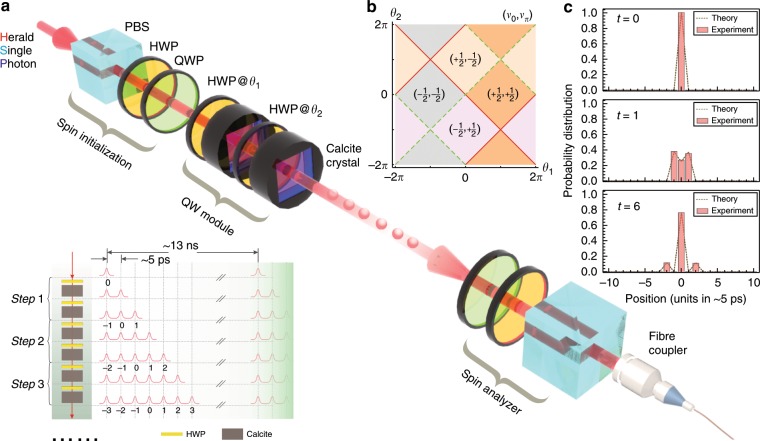
Sketch of the experimental setup. **a** Implementation of the time-multiplexing split-step quantum walk. A heralded single photon with a central wavelength of 780 nm generated from beam-like spontaneous parametric down conversion is adopted as the walker. The anticorrelation parameter^63^ measured in experiment is 0.031 ± 0.001. The polarization of the walker is prepared by a spin initialization module consisting of a PBS, HWP and QWP in sequence at the beginning and is measured by a spin analyzer consisting of a QWP, HWP and PBS in sequence at the end of the quantum walk. A full step of the split-step quantum walk is realized with two HWPs with their optical axes oriented at *θ*_1_ and *θ*_2_, respectively, for implementing the coin tossing, and two calcite crystals with their optical axes cut colinearly and orientated horizontally, for implementing the spin-orbit coupling. The final arrival time of the photon is measured by a homemade upconversion single-photon detector. In the inset of **a**, we show a diagram of the split-step quantum walk in the conventional time frame. To access the two shifted nonequivalent time frames, we change the rotation angle of the first HWP and add an extra HWP at the end for each complete step. The walker’s position space consists of the time bins (defining the arriving time of a single photon) with a pulse interval of 5 ps (determined by the length of the calcite crystal). The maximum repetition rate is 76 MHz, corresponding to a time of 13 ns. Here, we use ten quantum walk modules in total for a split-step quantum walk and one extra quantum walk module for adiabatically preparing some special initial states. **b** The complete topological phase diagram hidden in the split-step discrete-time quantum walk. In **c**, we present the probability distributions at times *t* = 0, *t* = 1 and *t* = 6 in the first configuration, i.e., the quench from a ground state of the Hamiltonian in the trivial phase (*θ*_2_ = *π*) and ending in a nontrivial phase (*θ*_1_ = 8*π*/9 and *θ*_2_ = −*π*/3). The total coincidence counts between the idle photon and the upconversion signal are above 200 Hz, and for each basis, we set the integration time to 200 s. PBS polarized beam splitter, HWP half-wave plate, QWP quarter-wave plate.

This work is carried out on our recently developed photonic discrete-time quantum walk platform based on a time-multiplexing protocol^38,43^. The critical operation in a discrete-time quantum walk is the conditional shift of the walker, which generates effective spin-orbit couplings^8^. Conventionally, in a photonic time-multiplexing quantum walk, this shift is implemented by optical loops^44^. Here, we use birefringent crystals that avoid extra loss appearing in conventional time-multiplexing schemes^12^. The experimental setup is sketched in Fig. 1a. Here, we employ the two orthogonal polarizations, horizontal and vertical, of the heralded single photon as the internal coin space, which is represented in the following as a pseudospin *μ* = ↑, ↓. We use two half-wave plates (HWPs) and two calcite crystals to implement a full split-step quantum walk^22–24^, as shown in Fig. 1a. That is, at each time, we repeat an identical sequence of four operations to manipulate the walker. First, a rotation $\hat R\left( {\theta _1} \right)$ in the internal pseudospin space with a tunable angle *θ*_1_ is realized via the first HWP. This rotation is followed by a conditional shift $\hat T_ \uparrow$ of the walker to the neighboring lattice site to the right provided its internal state is ↑, which is achieved through a birefringent crystal. Then, we perform another rotation $\hat R\left( {\theta _2} \right)$ with an angle *θ*_2_ and a further conditional shift $\hat T_ \downarrow$, where this time the walker moves one lattice site to the left provided its internal state is ↓. Probing the dynamics stroboscopically after each completed step of the quantum walk realizes a periodic Floquet evolution where the unitary time evolution operator $\hat U$ for one cycle is given by $\hat U\left( {\theta _1,\theta _2} \right) = \hat T_ \downarrow \hat R\left( {\theta _2} \right)\hat T_ \uparrow \hat R\left( {\theta _1} \right)$. In a time-multiplexing quantum walk, the discrete position space consists of time bins that stand for the arrival time of the walker and can be indexed by integers^44^. Initially, we prepare the photonic walker in a localized state on a given lattice site, e.g., *x* = 0 with a tunable superposition of ↑ and ↓ in the coin space. In our experimental realization, we can fully reconstruct the quantum state |Ψ_*t*_〉 in the subsequent evolution of the walker (see Methods)

1 $$\left| {{\mathrm{\Psi }}_t} \right\rangle = \mathop {\sum }\limits_{x,\mu } \psi _t\left( {x,\mu } \right)\left| {x\mu } \right\rangle$$

where $x \in {\Bbb Z}$ denotes the spatial point on the one-dimensional lattice and the quantum number *μ* = ↑, ↓ for the internal coin space. Accordingly, we achieve full experimental access to the state amplitudes $\psi _t\left( {x,\mu } \right)$ at each of the up to 10 time quench steps studied in this experiment, which is essential for the central goal of this work of dynamically characterizing quantum walks. The stroboscopic evolution of our periodically time-dependent system is determined by the associated Floquet Hamiltonian $\hat H_F\left( {\theta _1,\theta _2} \right)$ defined via $\hat U\left( {\theta _1,\theta _2} \right) = e^{ - iH_F\left( {\theta _1,\theta _2} \right)}$. For the split-step quantum walk, $\hat H_F\left( {\theta _1,\theta _2} \right) = {\int}_{ - \pi }^\pi {dk} H_F^k\left( {\theta _1,\theta _2} \right)$ is analogous to the Hamiltonian characterizing electrons in a solid with two bands, where *k* denotes the conserved lattice momentum^45,46^. From this perspective, this quantum walk can exhibit interesting topological properties in the sense that the corresponding ground state represents a topological insulator. As a natural periodically driven system, a complete classification of its topological phase needs to take into account the time frames, that is, the choice of the starting point^26–31^. In the split-step quantum walk, we have two nonequivalent times frames, i.e., $\hat U_1\left( {\theta _1,\theta _2} \right) = \sqrt {\hat R\left( {\theta _1} \right)} \hat T_ \downarrow \hat R\left( {\theta _2} \right)\hat T_ \uparrow \sqrt {\hat R\left( {\theta _1} \right)}$ and $\hat U_2\left( {\theta _1,\theta _2} \right) = \sqrt {\hat R\left( {\theta _2} \right)} \hat T_ \uparrow \hat R\left( {\theta _1} \right)\hat T_ \downarrow \sqrt {\hat R\left( {\theta _2} \right)}$. It is easy to check that the conventional time frame $\hat U$ defined above is equivalent to $\hat U_1$. The complete phase diagram of $\hat H_F\left( {\theta _1,\theta _2} \right)$ can then be given with the winding numbers defined in the two nonequivalent time frames^38^, which is shown in Fig. 1b. While quantum walks describe an inherently nonequilibrium dynamical process, signatures of these quasi-equilibrium topological properties have been observed experimentally, e.g., via the concomitant topological edge states^24^.

**Fig. 2:**
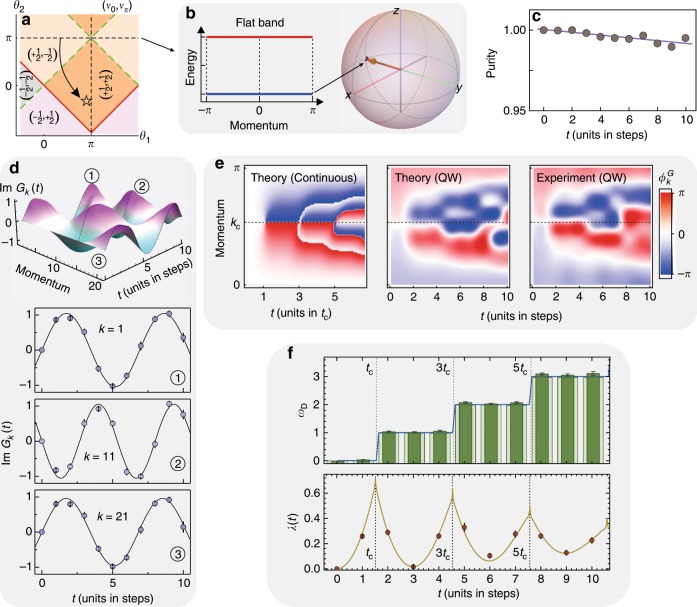
Experimental measurement of the DTOP for observing the DQPT. **a** The quenching strategy in terms of the phase diagram, starting from a ground state of the Hamiltonian with a flat band (*θ*_2_ = *π*) in the trivial phase and ending in a nontrivial phase (pentagram with *θ*_1_ = 8*π*/9 and *θ*_2_ = −*π*/3). The energy band (theoretical) and the initial state (black point for the theoretical expectation and arrow for the experiment) are presented in **b**. We fit the experimental data in rank 2 to reveal the decoherence, and the purity for each step is given in **c** (the errors are smaller than the point size). In **d**, we show how to extract the dynamical phase with full knowledge of the wavefunction for each step. The imaginary part of the Loschmidt amplitude is presented at the top, with three cases *k* = 1, *k* = 11 and *k* = 21 shown at the bottom. We read out the amplitude and period by fitting the measured results (circle points) to a trigonometric function. Density plots of the associated PGP $\phi _k^G\left( t \right)$ are shown in **e**, from left to right, for a theoretical consideration in momentum space (continuous-time evolution), theoretical simulation of the QW (discrete-time evolution) and our experimental results. The exact critical time is calculated from the continuous-time evolution, which is *t*_c_ = 1.513 and predicts the first occurrence of the DQPT. The experimentally measured DTOP is presented in **f** with the opaque bars; the blue line is the theoretical prediction numerically calculated in momentum space (continuously), and the transparent bars are the predictions from the simulation of the quantum walk. The vertical dashed lines show the critical times for each occurrence of the DQPT. At the bottom, we present the rate function *λ*(*t*) with the red line (obtained in the continuous simulation) and the experimental measured values with points. Each nonanalyticity predicts the occurrence of a DQPT. The errors are estimated using numerical Monte Carlo simulations considering the counting noise.

**Fig. 3:**
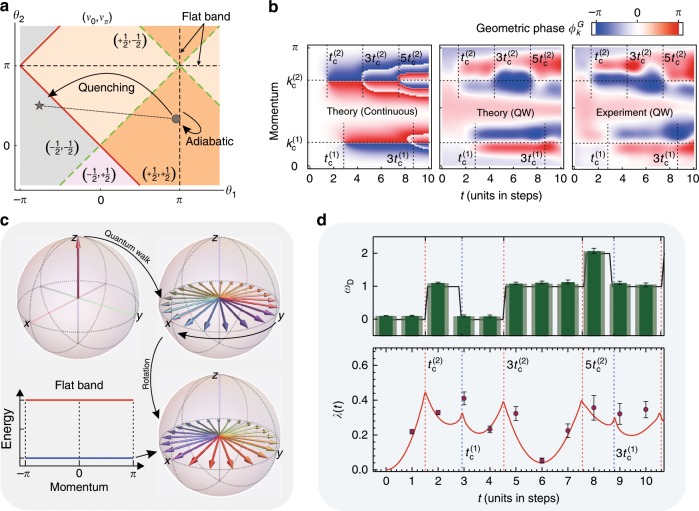
Observation of the DQPT in a quench between two different nontrivial topological phases. The strategy is shown in **a** in terms of the phase diagram. We start the quench in phase $\left( { + \frac{1}{2}, + \frac{1}{2}} \right)$ with *θ*_1_ = 8.6*π*/9 and *θ*_2_ = *π*/3 and end the quench in phase $\left( { - \frac{1}{2}, - \frac{1}{2}} \right)$ with *θ*_1_ = −7*π*/9 and *θ*_2_ = *π*/2. The initial state is prepared via adiabatic evolution starting from a ground state of the Hamiltonian with a flat band (*θ*_1_ = *π*). **c** The scheme for preparing the ground state of a Hamiltonian with a flat band in the topological nontrivial phase (details are given in Methods). **b** The PGP and **d** the DTOP. In this scenario, two critical times are observed. The rate function *λ*(*t*) is shown at the bottom of **d** with the experimental results indicated by points. The errors are estimated using numerical Monte Carlo simulations considering the counting noise.

The purpose of our present work is to go beyond such a quasi-equilibrium picture and characterize the dynamics of the quantum walk through a DTOP. To this end, we initially prepare the walker at *t* = 0 as a wave packet localized at *x* = 0 with $\left| {{\mathrm{\Psi }}_0} \right\rangle = \mathop {\sum}\nolimits_\mu {\psi _0} \left( {0,\mu } \right)\left| {0\mu } \right\rangle$. We choose the superposition in the coin space such that |Ψ_0_〉 represents a single-particle eigenstate in the lower of the two bands of an initial Floquet Hamiltonian $H_F^i$, which we can also implement dynamically in our setup. Preparing the ground state of $H_F^i$ is possible whenever $H_F^i$ exhibits flat bands, as it can be realized for the case where $H_F^i$ is topologically trivial or nontrivial; see Fig. 2b and Fig. 3c. Subsequently, we evolve the system according to the chosen split-step quantum walk characterized by *H*_*F*_, sequencing and monitoring the full nonequilibrium dynamics of the wavefunction. This protocol can be interpreted as a quantum quench from $H_F^i$ to *H*_*F*_, which, as detailed below, we can identify as a quench in a corresponding many-body system. Although the ground state of *H*_*F*_ cannot be reached in a quantum walk, from the observed dynamics of the DTOP, we obtain information about its topological properties.

### Dynamical topological order parameter

For the definition of the DTOP, it is essential that we have experimental access to the full amplitudes $\psi _t\left( {x,\mu } \right)$, including the phase information. In this sense, the proposed dynamical characterization relies crucially on the quantum nature of the quantum walk. The DTOP is defined through a lattice-momentum-dependent PGP $\phi _k^G\left( t \right)$, extending the concept of Berry’s geometric phase to nonadiabatic and noncyclic dynamics, which is naturally realized in our quantum walk experiment. Specifically, $\phi _k^G\left( t \right)$ measures the gauge invariant and geometric content of the acquired phase during the evolution at a given lattice momentum *k*. In formal terms, let us expand the state at a given time step *t* not in the real-space basis |*xμ*〉 as in Eq. (1) but rather in the lattice-momentum basis via $\vert{{\mathrm{\Psi}}_t}\rangle = {\int_{-\pi }^\pi} {dk}\vert{\psi_t} \left(k\right)\rangle$ with $\vert {\psi _t\left( k \right)}\rangle = {\sum \nolimits_\mu} {\psi _t} \left( {k,\mu } \right)\vert {k\mu }\rangle$ and $\psi _t\left( {k,\mu } \right)$, the Fourier transform of $\psi _t\left( {x,\mu } \right)$. The acquired phase $\phi _k\left( t \right)$ relative to the initial condition at a given *k* and time step *t* can be obtained from a polar decomposition of the Loschmidt amplitude ${\cal{G}}_k(t) = \langle\psi _0( k )\vert {\psi _t( k )} \rangle = r_k(t)e^{i\phi _k( t )}$. Importantly, $\phi _k\left( t \right)$ contains a gauge invariant part $\phi _k^G\left( t \right) = \phi _k\left( t \right) - \phi _k^{{\mathrm{dyn}}}\left( t \right)$, called the PGP, after subtracting a dynamical contribution, which in our case of a sudden quench is given by $\phi _k^{{\mathrm{dyn}}}\left( t \right) = - t\left\langle {\psi _t\left( k \right)} \right|H_F\left| {\psi _t\left( k \right)} \right\rangle$. With the acquired $\phi _k\left( t \right)$ and dynamical phase $\phi _k^{{\mathrm{dyn}}}\left( t \right)$, we can now define the DTOP *ω*_*D*_(*t*) as an integer-valued winding number associated with $\phi _k^G\left( t \right)$:

2 $$\omega _D\left( t \right) = \frac{1}{{2\pi }}\mathop {\int }\nolimits_0^\pi \frac{{\partial \phi _k^G\left( t \right)}}{{\partial k}} \in {\Bbb Z}$$

An analog of the DTOP in the context of quenched topological superconductors has been defined in ref. ^47^. The quantization of *ω*_*D*_ is imposed by particle-hole symmetry, which ensures that $\phi _{k = 0}^G\left( t \right) = \phi _{k = \pi }^G\left( t \right)$ and consequently $\phi _k^G\left( t \right)$ forms a loop on the unit circle^47^. Based on a full-state reconstruction of *ψ*_*t*_(*x*, *μ*), in our experiment, we measure the acquired phase *ϕ*_*k*_(*t*) and, importantly, the dynamical phase $\phi _k^{{\mathrm{dyn}}}\left( t \right)$, which allows us to map out the full momentum-dependent PGP $\phi _k^G\left( t \right)$; see Methods for technical details. In Fig. 2e, as an example of one realization of the split-step quantum walk, we show the experimentally obtained $\phi _k^G\left( t \right)$ along a trajectory of 10 time steps and compare the results with the theoretically expected values. For the first few time steps, the experimental data closely follow the ideal theoretical predictions. With the measured PGP $\phi _k^G\left( t \right)$ for each momentum, the integer-valued winding number *ω*_*D*_(*t*) can then be directly given by a simple Riemann sum according Eq. (2), as shown in Fig. 2f for instance. At later times, deviations become visible, which we trace back mainly to decoherence in the experiment leading to a reduction of the purity of the walker’s state and experimentally estimate in Fig. 2c. A loss of purity of only a few percent leads to substantial changes in the details of $\phi _k^G\left( t \right)$, highlighting the accuracy required both in the implementation of the unitary dynamics and in the state reconstruction. However, we find that as a dynamical topological quantum number, the DTOP *ω*_*D*_(*t*) is much more robust to a loss of purity, as shown below.

### Dynamical phase diagram of the split-step quantum walk

In the following, we use the observed integer-valued quantum number *ω*_*D*_(*t*) to dynamically characterize the realized quenched split-step quantum walk. The Floquet Hamiltonian before and after the quench is characterized by a doublet of topological invariants (*ν*_0_, *ν*_*π*_), where each invariant can take values of ±1/2 in our setup (as shown in Fig. 1b). When simply calling a Floquet Hamiltonian topologically trivial or nontrivial, we refer to the coarser ${\Bbb Z}_2$ classification obtained from the sign of the product *ν*_0_*ν*_*π*_, where ${\mathrm{sign}}\left( {\nu _0\nu _\pi } \right) = - 1$ signifies the trivial phase. We start by considering a setup where the initial condition of the walker implements an eigenstate of an associated topologically trivial Floquet Hamiltonian $H_F^i = H_F\left( {8\pi /9,\pi } \right)$ with $\left( {\nu _0,\nu _\pi } \right) = \left( { + 1/2, - 1/2} \right)$ and the subsequent time evolution is governed by a topologically nontrivial $H_F = H_F\left( {8\pi /9, - \pi /3} \right)$ with $\left( {\nu _0,\nu _\pi } \right) = \left( { + 1/2, + 1/2} \right)$; see Fig. 2a. Figure 2e shows the measured PGP $\phi _k^G\left( t \right)$ for this experimental sequence. With these geometric phases, we can further obtain the DTOP *ω*_*D*_(*t*), which is shown in Fig. 2f and closely follows the ideal theoretically expected values. For the first two time steps, the DTOP is consistent with *ω*_*D*_(*t*) = 0. Afterward, however, we observe a sudden jump of the DTOP to *ω*_*D*_(*t*) = 1; similarly, at later times, the DTOP jumps to *ω*_*D*_(*t*) = 2. Since *ω*_*D*_(*t*) is a quantized integer, this change in *ω*_*D*_(*t*) can only occur in a nonanalytic fashion, which is indicative of behavior that is typically associated with phase transitions. Below, we show that such a relation to a dynamical analog of a phase transition can indeed be established.

**Fig. 4:**
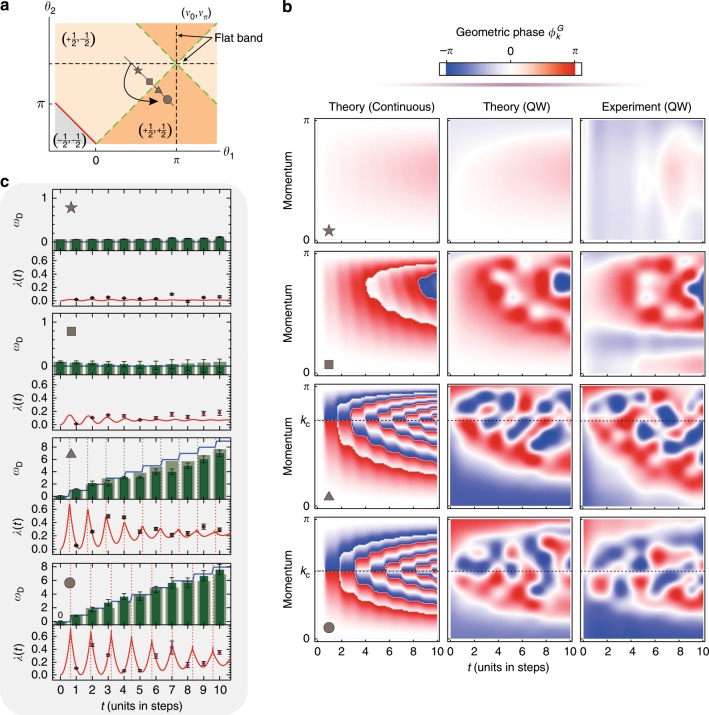
Measurement of the DTOP for determining the phase boundary. **a** The quenching strategies in terms of the phase diagram: we start the quench from the flat band (*θ*_2_ = *π*) and end the quench with different selected Hamiltonians, two Hamiltonians still in the trivial phase and two Hamiltonians in the nontrivial phase for a comparison; *θ*_1_ = 5*π*/9 and *θ*_2_ = 8*π*/9 for the pentagram, *θ*_1_ = 6*π*/9 and *θ*_2_ = 7*π*/9 for the square, *θ*_1_ = 7*π*/9 and *θ*_2_ = 6*π*/9 for the diamond, and *θ*_1_ = 8*π*/9 and *θ*_2_ = 5*π*/9 for the circle, which form a line crossing the phase boundary. **b** The density plots of the corresponding PGP for each case. In **c**, we show the corresponding rate function *λ*(*t*) and the DTOP *ω*_*D*_ as a function of time. The lines are predicted in the continuous calculation. The points and opaque bars are the experimental results. The transparent bars are predictions from the simulation of the quantum walk. The errors are estimated using numerical Monte Carlo simulations considering the counting noise.

We now study the dynamics of the quantum walk not only for a fixed parameter set but also along a line in parameter space upon keeping the initial condition fixed as specified in Fig. 4a. The time evolution of the DTOP for the different sets (*θ*_1_, *θ*_2_) is shown in Fig. 4c. For *θ*_1_ = 5*π*/9 and *θ*_2_ = 8*π*/9, which is indicated by a star and closest in terms of distance to the initial condition, we observe that the DTOP *ω*_*D*_(*t*) = 0 vanishes along the full trajectory. We also find that the same behavior for *θ*_1_ = 6*π*/9 and *θ*_2_ = 7*π*/9 (square symbol) represents a qualitatively different dynamical regime than the case observed in Fig. 2f. However, as soon as our parameter quench crosses the boundary between the two Floquet regimes characterized by $\left( {\nu _0,\nu _\pi } \right) = \left( { + 1/2, - 1/2} \right)$ and $\left( {\nu _0,\nu _\pi } \right) = \left( { + 1/2, + 1/2} \right)$, we recover the jumps in *ω*_*D*_(*t*) at successive times with an overall monotonously increasing envelope for the next parameter sets *θ*_1_ = 7*π*/9 and *θ*_2_ = 6*π*/9 (triangle symbol) and *θ*_1_ = 8*π*/9 and *θ*_2_ = 5*π*/9 (circle symbol), respectively. According to these observations, at this point, we can identify two qualitatively different dynamical phases as characterized by the temporal behavior of the DTOP.

We find, however, that there exists also a third phase characterized by yet another behavior of *ω*_*D*_(*t*). For observing this behavior, we study the DTOP for a different initial condition for which the hypothetical ground state of the Floquet Hamiltonian $H_F^i$ would be of topological nature with $\left( {\nu _0,\nu _\pi } \right) = \left( { + 1/2, + 1/2} \right)$; see Fig. 3a. Upon time evolution with *H*_*F*_ corresponding to a different topological phase, we again observe that the DTOP changes its value at a sequence of points in time. Different from the previous cases, however, we observe that the DTOP can behave nonmonotonously over time. By drawing an analogy between the realized quantum walk and an equivalent quantum many-body problem, we explain the three observed dynamical phases in terms of a DQPT below.

**Fig. 5:**
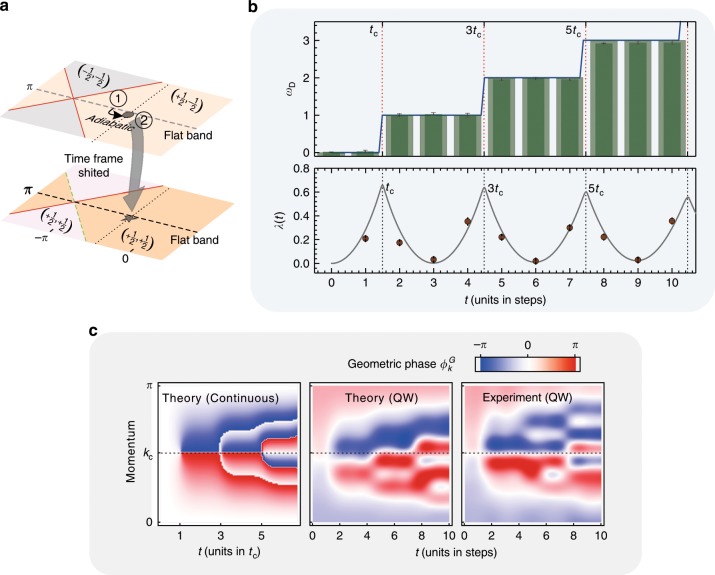
Observation of the DQPT in a quench by shifting the time frame. The strategy is presented in **a** in terms of the phase diagram. We initialize the system in a ground state of a given Hamiltonian *H*(*θ*_1_, *θ*_2_) with *θ*_1_ = −*π*/3, *θ*_2_ = 8.6*π*/9, which is located in the trivial phase. We adopt adiabatic evolution starting from the flat band again to prepare the initial state. With the time frame shifted, the winding of the eigenvectors of the given Hamiltonian changes its features, from zero to ±1. We show the DTOP in **b** and the PGP in **c**. The errors are estimated using numerical Monte Carlo simulations considering the counting noise.

For a complete classification of a periodically driven system, it is important to consider different time frames^26–31^. To this end, besides the conventional quench realized by sudden changes in the control parameter *θ*_1_ or *θ*_2_, we now consider a modified quench protocol, i.e., a quench induced by a sudden change of the time frame (see Fig. 5a for an illustration). First, we fix the time frame in $\hat U_1$. By performing an adiabatic evolution starting from the origin with the spinor state |↓_*y*_〉 (which is the superposition state of the lower band states for quantum walks with the parameters constrained on the dashed line in the trivial phase as shown in Fig. 5a), the system can be further initialized in the superposition state of the lower band states of a more general quantum walk with the Hamiltonian $H_{{\mathrm{eff}}}\left( { - \pi /3,8.6\pi /9} \right)$. (Note this quantum walk is still in the trivial phase.) Then, we suddenly change the time frame from $\hat U_1$ to a nonequivalent time frame $\hat U_2$ (ref. ^38^) while keeping the parameters unchanged. Nevertheless, the effective Hamiltonians *H*_*F*_ changes with different topological invariants upon changing time frames. The experimental results for this scenario are shown in Fig. 5b, c. Again, we observe characteristic behavior of the DTOP, monotonously increasing with time, which corresponds to the dynamical phase shown in Figs. 2f and 4c, as expected.

### Dynamical quantum phase transitions

The real-time nonanalytic behavior of the DTOP enables an intriguing analogy with the phenomenon of DQPTs^48,49^, which allows us to explain our observations in light of an equivalent many-body problem. To this end, we map our quantum walk system, for which the state is given by a coherent superposition $| {{\mathrm{\Psi }}_t} \rangle = {\int}_{ - \pi }^\pi {dk} | {\psi _t} ( k )\rangle$ of lattice-momentum modes (Bloch states), to a fermionic many-body system, whose state is given by a Slater determinant of |*ψ*_*t*_(*k*)〉. We note that this mapping requires complete access to |*ψ*_*t*_(*k*)〉, which we can achieve in our setup in parallel due to the large degree of coherence. Within the theory of DQPTs, the central object is the Loschmidt amplitude ${\cal{G}}\left( t \right)$, which for the corresponding many-body system factorizes as ${\cal{G}}\left( t \right) = \mathop {\prod}\nolimits_k {{\cal{G}}_k} \left( t \right)$. DQPTs are hallmarked by nonanalytic points in time of the associated rate function $g\left( t \right) = - N^{ - 1}{\mathrm{log}}\left[ {{\cal{G}}\left( t \right)} \right]$, which plays the role of a formal analog to a free energy density. Here, *N* denotes the number of degrees of freedom, i.e., the number of involved lattice-momentum modes. Such DQPTs and their signatures have been recently observed in various systems^50–57^.

In all the figures, we have included a theoretical calculation of ${\uplambda}\left( t \right) = 2{\mathrm{Re}}\left[ {g\left( t \right)} \right]$ for the many-body system equivalent to the respective implemented quantum walk. For example, in Fig. 2f, the situation corresponds to a quantum quench in a two-band fermionic system from an initial topologically trivial insulating state, the ground state of $H_F^i$, to a final Hamiltonian *H*_*F*_ exhibiting topologically nontrivial properties.

Using the analogy with the equivalent many-body system, we can further relate the equilibrium properties of the Floquet Hamiltonian *H*_*F*_ to the dynamics of the DTOP observed for the quantum walk. First, it is shown that a jump in the DTOP always occurs with a DQPT in the considered systems^48,49^. This is indeed what we find in our experiment. The times where the observed DTOP changes its topologically quantized value coincide with the critical times at which the corresponding many-body system undergoes a DQPT, as hallmarked by a logarithmic singularity in *g*(*t*).

All potential DQPTs that can occur in the considered models can be grouped into two classes, enabling an overall classification in terms of three dynamical phases, with the third phase being the dynamics without the occurrence of a DQPT yielding *ω*_*D*_(*t*) = 0. First, DQPTs have to occur whenever the initial and final Hamiltonians, here $H_F^i$ and $H_F$, are topologically inequivalent in the ${\mathbb Z}_2$ classification corresponding to a positive or negative sign of *ν*_0_*ν*_*π*_^58^, where *ν*_0_*ν*_*π*_ > 0 refers to the topological phase and *ν*_0_*ν*_*π*_ < 0 refers to the trivial phase, respectively. In this sense, these DQPTs are topologically protected, and their data are shown in Fig. 2. Second, DQPTs can be accidental, without changing the product *ν*_0_*ν*_*π*_, thus leaving the ${\mathbb Z}_2$ classification of the static system unchanged. Notably, in our present Floquet context, such accidental DQPTs occur precisely when both *ν*_0_ and *ν*_*π*_ switch signs while leaving their product unchanged. This scenario gives a clear topological meaning to this second kind of DQPT in our split-step quantum walk setup. Remarkably, the DTOP observed in this work is capable of qualitatively distinguishing these different kinds of DQPT scenarios (cf. Figs. 2 and 3).

## Discussion

In this experiment, we have achieved a dynamical characterization of split-step quantum walks using a DTOP—an integer-valued quantum number that measures the winding of the geometric phase in the lattice-momentum space. The possibility to reconstruct the full wavefunction of the quantum walk state with access to the full set of quantum amplitudes, including their phase information, has been central for our measurement of the DTOP. Our results clearly show that as a global quantum number of the system’s dynamical topological phase, the DTOP is robust with respect to decoherence in our experimental platform on the time scales studied in this work. The robustness of the DTOP to disorder^59^ might deserve further experimental investigations in the future. With a mapping onto a quantum quench in an equivalent quantum many-body problem, we have shown that this dynamical characterization is intimately related to the phenomenon of DQPTs in the unitary real-time evolution. In this way, we provide a nonequilibrium perspective of quantum walks, which can be understood as a starting point for approaching time-dependent processes from an inherently dynamical angle that goes beyond the notion of equilibrium statistical physics. With this perspective and by mapping onto quenches in an equivalent quantum many-body system, our experiment offers a versatile platform for the study of the coherent nonequilibrium dynamics of many paradigmatic models such as the Su-Schrieffer-Heeger model^45^, the p-wave Kitaev chain^60^, or the transverse field Ising model^61^ in the future. We expect that our method to be straightforwardly extended to other photonic systems, such as continuous-time quantum walks based on integrated photonics^62^.

## Materials and methods

### Initial state preparation

Before starting the quantum walks, we prepare the system initially in a single-particle eigenstate of an effective Floquet Hamiltonian $H_F^i$, which we can finally associate with a quantum quench in an equivalent quantum many-body problem. We proceed by distinguishing three different cases for $H_F^i$: (a) a trivial flat-band Hamiltonian, (b) a topologically nontrivial flat-band Hamiltonian and (c) a general Hamiltonian without flat bands. For (a), the ground state of the flat band can be localized on a single site at the origin in real space with the spin pointing in the *y*-direction, e.g., $\left| {{\mathrm{\Psi }}_0} \right\rangle = \left| {x = 0 \downarrow _y} \right\rangle$ for $H_F^i\left( {\theta _1^i,\pi } \right)$. The situation in scenario (b) is slightly more complicated. We first initialize the system in the state $\left| {x = 0 \uparrow } \right\rangle$. Then, we perform a full quantum walk step with the parameters (*π*, *π*/2); finally, we perform an additional spin rotation along the *σ*_2_ axis with an angle *π*/2 (see Fig. 3c). In this way, the system is prepared in the state $\left( {\left| { - 1 \uparrow } \right\rangle - i\left| {0 \downarrow } \right\rangle } \right)/\sqrt 2$, which, in its momentum space representation, corresponds to a superposition including all of the single-particle states in the lower band of the nontrivial flat-band Hamiltonian $H_F^i\left( {\pi ,\theta _2^i} \right)$. Case (c) is important for effectively realizing quantum quenches between two inequivalent nontrivial Hamiltonians $H_F^i$ and *H*_*F*_ in the equivalent many-body problem and for a quantum quench driven by a change of time frame. To achieve an initial state corresponding to a nonflat-band Hamiltonian, we start from a flat-band ground state according to (a) or (b). Then, we perform an additional step to adiabatically transfer the system into the ground state of a general target Hamiltonian with the same phase, which is always possible due to the finite energy gap.

### Full state reconstruction

Our new platform for implementing quantum walks allows us to access the full wavefunction, including the phase information (see ref. ^38^ for a detailed discussion). In brief terms, suppose that the system after *t* steps of the quantum walk is in state |Ψ_*t*_〉 (see Eq. 1). We then carry out three steps to obtain the complex coefficients *ψ*_*t*_(*x*, *μ*): First, we perform a local projection measurement on the spin for each site and obtain a count set *S*. Then, after shifting all of the spin-up components a step backward (by inserting an additional birefringent crystal), we perform a local projection measurement on the spin again and obtain another count set $\tilde S$. Finally, based on a simulated annealing algorithm, we carry out a numerical global program to find an optimal state of the form given in Eq. (1), which reproduces the two count sets $S,\tilde S$ with the largest probability. As the number of projection bases equals 4(2*N* − 1), with *N* being the lattice size, which is much greater than the number of independent variables 2(2*N* − 1) in the wavefunction in Eq. (1), we can systematically improve the rank of the target state and monitor the decoherence in the experiment. With full knowledge of |Ψ_*t*_〉, i.e., both the amplitudes and phases of the coefficients *ψ*_*t*_(*x*, *μ*), we can readily obtain the wavefunction in the momentum representation by performing a Fourier transform. Concretely, we perform a discrete Fourier transform separately on the spin-up and spin-down components and then renormalize the components for each quasimomentum. The decoherence in our system resulting in a degeneration of the purity can be estimated by increasing the rank of the target density matrices (the pure state situation corresponds to rank 1). The results for the rank 2 scenario are shown in Fig. 2c.

### Measuring the Pancharatnam geometric phase

We now provide details on how the PGP, which is at the heart of our present study, can be directly extracted from our experimental data. We focus on the PGP $\phi _k^G$ associated with a fixed lattice momentum *k*, defined via ${\cal{G}}_k\left( t \right) = \left\langle {\psi _0\left( k \right)|\psi _t\left( k \right)} \right\rangle = r_k\left( t \right)e^{i\phi _k\left( t \right)}$ with $\phi _k\left( t \right) = \phi _k^G\left( t \right) + \phi _k^{{\mathrm{dyn}}}\left( t \right)$. Our direct observation of $\phi _k^G$ then results from the independent observation of the total phase $\phi _k\left( t \right)$ and the dynamical phase $\phi _k^{{\mathrm{dyn}}}\left( t \right)$ of the time-evolved wavefunction $\left| {\psi _t\left( k \right)} \right\rangle$ relative to the initial condition $\left| {\psi _t\left( 0 \right)} \right\rangle$. The total phase is an immediate result of the full-state tomography of the time-evolved wavefunction. To isolate the dynamical phase $\phi _k^{{\mathrm{dyn}}}\left( t \right)$, we expand the initial state $\left| {\psi _t\left( 0 \right)} \right\rangle = g_k\left| {u_k^ - } \right\rangle + e_k\left| {u_k^ + } \right\rangle$ in the eigenbases $\left| {u_k^ \pm } \right\rangle$ of the final Hamiltonian *H*_*F*_ with $\pm {\it{\epsilon }}_k^f$ denoting the corresponding eigenenergies. In this representation, the Loschmidt amplitude takes the form ${\cal{G}}_k\left( t \right) = \left( {\left| {g_k} \right|^2 + \left| {e_k} \right|^2} \right){\mathrm{cos}}\left( {{\it{\epsilon }}_k^ft} \right) + i\left( {\left| {g_k} \right|^2 - \left| {e_k} \right|^2} \right){\mathrm{sin}}\left( {{\it{\epsilon }}_k^ft} \right)$. By observing the amplitude and phase of the oscillations of ${\cal{G}}_k\left( t \right)$, we hence obtain $\left( {\left| {g_k} \right|^2 - \left| {e_k} \right|^2} \right)$ and ${\it{\epsilon }}_k^f$, respectively; see Fig. 2d. The acquired $\left( {\left| {g_k} \right|^2 - \left| {e_k} \right|^2} \right)$ and ${\it{\epsilon }}_k^f$ determine the dynamical phase $\phi _k^{{\mathrm{dyn}}}\left( t \right) = {\it{\epsilon }}_k^ft\left( {\left| {g_k} \right|^2 - \left| {e_k} \right|^2} \right)$ and thus the PGP $\phi _k^G\left( t \right) = \phi _k\left( t \right) - \phi _k^{{\mathrm{dyn}}}\left( t \right)$. It should be noted here that to perform such a fitting, we should sample the Loschmidt amplitude at least over one period. Generally, without considering decoherence, we obtain a higher quality fit for a larger number of time steps. To obtain a clear experimental signature of the quantization of the DTOP and the transition between these discrete values, we must ensure that the plateaus of the constant value of the DTOP extend at least over a range of three discrete time steps. While our system can support much larger scale quantum walks^38^, to achieve a balance between the experimental challenge and the clarity of the phenomena, here we perform 10 full time steps, covering a 1.5 oscillation period, as shown in Fig. 2d, which are sufficient for extracting the dynamical phase.
